# The Potent and Selective Histamine H3 Receptor Antagonist E169 Counteracts Cognitive Deficits and Mitigates Disturbances in the PI3K/AKT/GSK-3β Signaling Pathway in MK801-Induced Amnesia in Mice

**DOI:** 10.3390/ijms241612719

**Published:** 2023-08-12

**Authors:** Sabna Abdalla, Nermin Eissa, Petrilla Jayaprakash, Rami Beiram, Kamil J. Kuder, Dorota Łażewska, Katarzyna Kieć-Kononowicz, Bassem Sadek

**Affiliations:** 1 Department of Pharmacology & Therapeutics, College of Medicine and Health Sciences, United Arab Emirates University, Al Ain P.O. Box 15551, United Arab Emirates; 202070050@uaeu.ac.ae (S.A.); petrilla.jp@uaeu.ac.ae (P.J.); rbeiram@uaeu.ac.ae (R.B.); 2 Zayed Bin Sultan Center for Health Sciences, United Arab Emirates University, Al Ain P.O. Box 15551, United Arab Emirates; 3 Department of Biomedical Sciences, College of Health Sciences, Abu Dhabi University, Abu Dhabi P.O. Box 59911, United Arab Emirates; nermin.eissa@adu.ac.ae; 4 Department of Technology and Biotechnology of Drugs, Faculty of Pharmacy, Jagiellonian University Medical College in Kraków, Medyczna 9 St., 30-688 Krakow, Poland; kamil.kuder@uj.edu.pl (K.J.K.); dorota.lazewska@uj.edu.pl (D.Ł.); mfkonono@cyf-kr.edu.pl (K.K.-K.)

**Keywords:** histamine H3 receptors, antagonists, E169, MK801-induced amnesia, PI3K/AKT/GSK-3β signaling pathway, novel object recognition test, memory, cognition, neuroinflammation, neurodegenerative diseases, Alzheimer’s disease, mice

## Abstract

The role of histamine H3 receptors (H3Rs) in memory and the prospective of H3R antagonists in pharmacological control of neurodegenerative disorders, e.g., Alzheimer’s disease (AD), is well-accepted. Therefore, the procognitive effects of acute systemic administration of H3R antagonist E169 (2.5–10 mg/kg, i.p.) on MK801-induced amnesia in C57BL/6J mice using the novel object recognition test (NORT) were evaluated. E169 (5 mg) provided a significant memory-improving effect on MK801-induced short- and long-term memory impairments in NORT. The E169 (5 mg)-provided effects were comparable to those observed with the reference phosphatidylinositol 3-kinase (PI3K) inhibitor LY294002 and were abrogated with the H3R agonist (*R*)-α-methylhistamine (RAMH). Additionally, our results demonstrate that E169 ameliorated MK801-induced memory deficits by antagonism of H3Rs and by modulation of the level of disturbance in the expression of PI3K, Akt, and GSK-3β proteins, signifying that E169 mitigated the Akt-mTOR signaling pathway in the hippocampus of tested mice. Moreover, the results observed revealed that E169 (2.5–10 mg/kg, i.p.) did not alter anxiety levels and locomotor activity of animals in open field tests, demonstrating that performances improved following acute systemic administration with E169 in NORT are unrelated to changes in emotional response or in spontaneous locomotor activity. In summary, these obtained results suggest the potential of H3R antagonists such as E169, with good in silico physicochemical properties and stable retained key interactions in docking studies at H3R, in simultaneously modulating disturbed brain neurotransmitters and the imbalanced Akt-mTOR signaling pathway related to neurodegenerative disorders, e.g., AD.

## 1. Introduction

**Figure 1:**
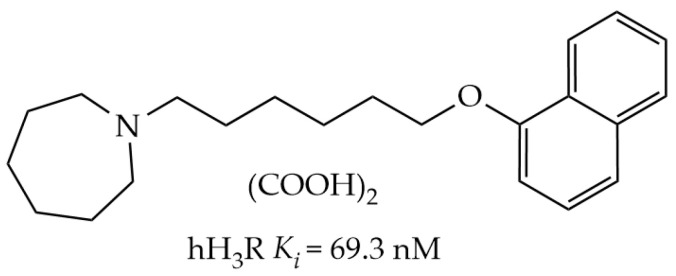
Chemical structure and affinity for human histamine H_3_ receptor of E169.

The core representative character of Alzheimer’s disease (AD) as a neurodegenerative disease is the progressive decline in cognitive performance [1,2,3]. The challenges associated with the development of potential therapies for AD are due to the complicated pathophysiology of AD including numerous pathways, e.g., defective β-amyloid protein metabolism, and abnormalities in central neurotransmissions for acetylcholine (ACh), glutamate (Glu), noradrenaline, serotonin (5-HT), and dopamine (DA), and the association of AD with inflammatory and/or oxidative and hormonal pathways [4,5,6]. Notably, in the past two decades, there has been development in research regarding the role of brain histamine on behaviors in both physical and mental disorders, such as AD, schizophrenia, and memory deficits [7,8]. By blocking centrally located histamine H3 receptors (H3Rs), which causes an increment in the release of brain neurotransmitters such as HA, ACh, DA, and norepinephrine, preclinical research suggested that H3R antagonists could potentially play a role in the therapeutic management of a variety of cognitive disorders, such as memory deficit, attention-deficit/hyperactivity disorder (ADHD), AD, and schizophrenia. Importantly, in 2016, the European Medical Agency approved a marketing application for pitolisant (WakixR) as an orphan drug for narcolepsy and in 2021 for obstructive sleep apnea (OzawadeR) [8]. Recently, cognitive disorders have been related to neuroinflammation, as this immunogenic response in the brain occurs mainly due to microglia which are resident macrophages of the CNS with various essential functions, including neuronal well-being and normal function and neuroprotection [9,10]. As a result, ongoing activation of the innate and adaptive immune system is a key factor in the development of several dementias and AD [11,12,13]. Moreover, the PI3K/AKT/GSK-3β signaling pathway was found to be involved in the progression of numerous neurodegenerative disorders including AD [14]. Accordingly, a lot of evidence suggested that activation of microglia and the PI3K/AKT/GSK-3β signaling pathway enhances the expression of several inflammatory cytokines and proteins [15]. Based on the aforementioned preclinical observations, the central H3Rs represent a possible target for developing novel agents with a potential therapeutic role in neuropsychiatric multi-neurotransmitter disorders, e.g., AD. First, ADMET properties of E169 and its docking to the human H3R receptor were calculated in silico. Next, E169 was tested for its memory-enhancing effects on MK801-induced memory deficits in adult male mice through conducting novel object recognition tests (NORT). Hence, the newly developed highly potent non-imidazole H3R ligand E169 [1-(6-(naphthalen-1-yloxy)hexyl)azepane], [16] (Figure 1) was selected for in vivo studies, as this test compound exhibited good in silico physicochemical properties and stably retained key interactions in docking studies at H3R. Additionally, the chemical synthesis of E169 is easy to handle in a one-step reaction with high yield which is again an additional advantage when it comes to further drug development.

Additionally, and to exclude any confounding factors, the effects of E169 on locomotor activity and anxiety-like behavior of the same animals were tested in open field tests (OFT). Moreover, the mitigating effects of E169 and the phosphatidylinositol 3-kinase (PI3K) inhibitor LY294002 on the level of disturbance in the expression of PI3K, Akt, and GSK-3β proteins in the hippocampus of treated mice were assessed. Furthermore, the abrogative effects of the co-administered centrally acting histamine H3R agonist (*R*)-α-methylhistamine (RAMH) on the E169-provided memory-enhancing effects in NORT and mitigating levels of PI3K, Akt, and GSK-3β protein’s expression in hippocampus were assessed.

## 2. Results

### 2.1. In Silico Evaluation of E169

#### 2.1.1. In Silico ADMET and Drug-Likeness Properties

ADMET parameters were calculated in the online programs SwissADME, ProToxII, pkCSM, and Biotransformer (http://www.swissadme.ch, accessed on 4 January 2023; https://tox-new.charite.de, accessed on 4 January 2023; https://biosig.lab.uq.edu.au/pkcsm, accessed on 4 January 2023; http://biotransformer.ca/new, accessed on 4 January 2023). The results are presented in Tables S1–S6 in the supplementary material. The compound has good physicochemical properties, with the exception of a slightly high predicted lipophilicity (mean log P = 5.12). This value is higher than the recommended value for compounds that should easily cross the blood–brain barrier (log P approx. 2), but both SwissAdme and pkCSM predict good permeation of this compound across the BBB barrier. Both also predict good absorption from the gastrointestinal tract. In addition, SwissAdme and pkCSM predict the possibility of interaction with cytochrome P450, in particular its isoforms CYP1A2 and CYP2D6, as an inhibitor of these enzymes. Further, pkCSM suggests the possibility of this molecule being a substrate for CYP3A4. The metabolism proposed by the Biotransformer program predicts hydroxylation at different positions in the alkyl chain and the alpha-naphthyl ring. The results of the calculations show that E169 has good drug-likeness parameters based on four well-known rules (Lipnsky, Gosh, Veber, and Egan). Only the Muegge rule does not predict drug likeness. The ProtoxII program predicts immunotoxicity while the pkCSM program predicts mutagenicity. In contrast, both programs do not predict hepatotoxicity.

#### 2.1.2. In Silico Docking Studies

**Figure 2:**
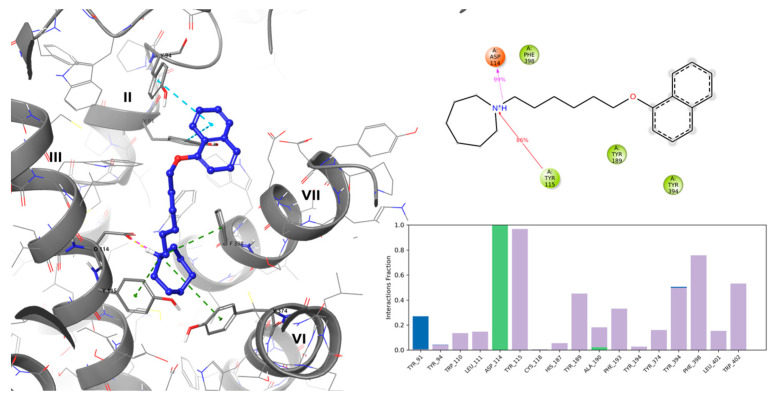
(**Left panel**): Putative binding mode of E169 (**left**) in the histamine H_3_R receptor binding site. Yellow dashed lines express hydrogen bonds, magenta—salt bridges, green—cation–π interactions, blue—π–ππ interactions. Roman numbers denote respective TMs. (**Right panel**): MD simulation of ligand–protein contact summary (**top**), and contacts histogram (**bottom**; green—H-bond, violet—hydrophobic contact, blue—water bridges).

A recent publication of H3R complexed with an antagonist (PDB ID: 7F61 [17] allowed for confirmation of putative ligand–receptor interactions, as well as for a better understanding of the receptor function itself. For the present study, the structure of E169 was chosen. The compound was characterized by relatively high dG bind value of —108.24 kcal/mol and occupied the H3R binding pocket in similar mode to that of the resolved complex ligand PF03654746, preserving a crucial histamine H3R antagonist/inverse agonist interaction, namely salt bridge and/or hydrogen bond formation between protonated amine nitrogen and ASP114^3.32^ (superscripts denote Ballesteros–Weinstein numbering) [18]. Additional west-end stabilization of the ligand through cation–π interactions, with caging the aromatic sidechains of Y115^3.33^, F398^7.39^, and Y374^6.51^, was also found. The east-end naphthalene substituent of E169 occupied the space fenced by aromatic features of TYR189 (ECL2) on the top, and TYR91^2.61^ and TYR94^2.64^ on the sides that also stabilized the structure through π–π stacking interactions (Figure 2).

**Figure 3:**
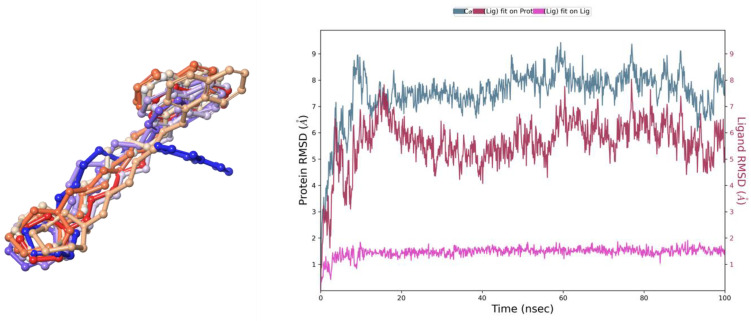
(**Left panel**): Trajectory snapshots of E169 in a 100 ns MD simulation. Each color depicts a different frame: 0 ns blue, then through the violet spectrum (dark to light: 10–40 ns) to grey (50 ns) and vice versa through the orange spectrum (light to dark: 60–90 ns) to red (100 ns). (**Right panel**): Time evolution of RMSD for ligand (magenta) and protein (grey-blue and dark red) for particular frames with respect to a reference frame at 0 ns.

The stability of the calculated pose was further evaluated by means of 100 ns molecular dynamics (MD) simulations. From the simulation, 10 snapshots were selected (starting position and after every 10 ns, Figure 3). Considering only the ligand position during the simulation, it can be observed that E169 was stable through the simulation, retaining key interactions (Figure 2), followed by a very consistent set of interacting amino acids. However, at the beginning of the simulation (~5 ns) a bi-aromatic moiety twisted from TM7 towards the extracellular space, resulting in an overall higher ligand RMSD value. Nevertheless, this conformational change allowed for additional stabilization from time to time by Y3947.35 and F193 (ECL2).

### 2.2. E169 Ameliorated MK801-Induced Short-Term Memory Deficits in C57BL/6J Mice

**Figure 4:**
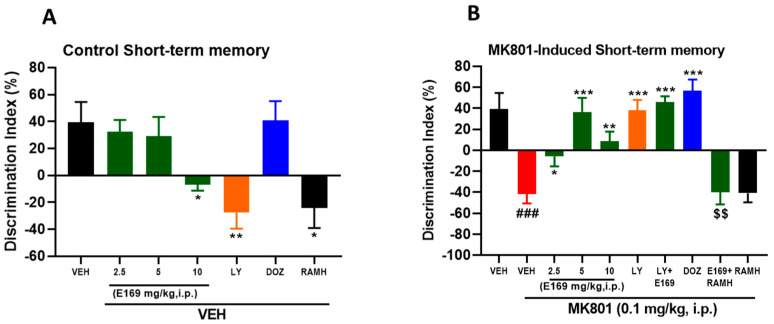
E169 ameliorated MK801-induced short-term memory deficits in C57BL/6J mice. The columns represent the means ± SEM (n = 6). (**A**) Observed DI values of control mice. (**B**) Observed DI values of MK801-induced STM deficits in mice. (**A**) * *p* < 0.05; ** *p* < 0.01 vs. VEH-treated control mice. (**B**) * *p* < 0.05; ** *p* < 0.01; *** *p* < 0.001 vs. MK801-treated mice. ^###^ *p* < 0.001 vs. VEH-treated mice; ^$$^ *p* < 0.01 vs. E169 (5 mg/kg) + MK801-treated mice.

Figure 4A shows that there was a significant reduction in Discrimination Index (DI) in the group that was injected with MK801 (F(1,10) = 28.50, *p* < 0.001) in comparison to the naive mice group. Moreover, there was a significant increase in DI in MK801-treated mice following systemic injection with E169 (2.5, 5, and 10 mg/kg, i.p.), with (F(1,10) = 7.67, *p* < 0.05), (F(1,10) = 23.98, *p* < 0.001), and (F(1,10) = 15.24, *p* < 0.01), respectively, compared to MK801-treated mice. Furthermore, MK801-treated mice treated with LY294002 (7.5 mg/kg) exhibited a considerable increase in DI value compared to the MK801-treated mice (F(1,10) = 36.13, *p* < 0.001), demonstrating the memory-enhancing effect of LY294002 on MK801-induced deficits in mice. In addition, amnesic mice injected with LY294002 and E169 (5 mg/kg, i.p.) exhibited a considerable increase in the observed DI (F(1,10) = 28.76, *p* < 0.001) in comparison to the MK801-treated mice group, and no significance (F(1,10) = 0.018, *p* = 0.89) was observed between the groups treated with LY (7.5 mg/kg, i.p.) and E169 (5 mg/kg, i.p.) (Figure 4B), demonstrating no synergistic effects of combining E169 with LY on MK801-induced short-term memory deficits in mice. In an additional experimental study, co-administration of the CNS-penetrant H3R agonist RAMH (10 mg/kg, i.p.) reversed E169-provided memory-enhancing effects as observed in the reduced DI value compared with MK801-mice treated with only E169 (5 mg/kg) with (F(1,10) = 17.82, *p* < 0.01). Notably, there was no significant difference between the control groups treated with E169 (2.5 or 5 mg/kg), with (F(1,10) = 0.17, *p* = 0.68) and (F(1,10) = 0.24, *p* = 0.62), respectively, and the VEH-treated control group (Figure 4A). However, there was a significant decrease in the observed DI value for control mice treated with the higher doses of E169 (10 mg/kg, i.p.) with (F(1,10) = 8.60, *p* < 0.05), which could be explained with off-target effects of E169 (Figure 4A).

Interestingly, the control mice group treated with LY294002 (7.5 mg/kg, i.p.) exhibited a considerable and significant decrease in the obtained DI value (F(1,10) = 12.12, *p* < 0.01) in comparison with VEH-treated control mice, demonstrating the negative effects of LY294002 on the overall behaviors of naïve mice (Figure 4A). Moreover, control mice that were treated with RAMH (10 mg/kg, i.p.) showed a significant reduction in the observed DI value (F(1,10) = 8.97, *p* < 0.05) compared with VEH-treated control mice (Figure 4A).

### 2.3. E169 Ameliorated MK801-Induced Long-Term Memory Deficits in C57BL/6J Mice

**Figure 5:**
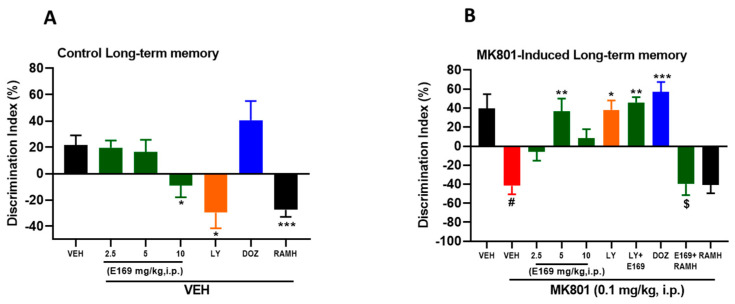
E169 ameliorated MK801-induced long-term memory deficits in C57BL/6J mice. The columns represent the means ± SEM (n = 6). (**A**) Observed DI values for control mice. (**B**) Observed DI values on MK801-induced STM deficits in mice. (**A**) * *p* < 0.05; *** *p* < 0.01 vs. VEH-treated control mice. (**B**) * *p* < 0.05; ** *p* < 0.01; *** *p* < 0.001 vs. MK801-treated mice; ^#^ *p* < 0.05 vs. VEH-treated mice ^$^ *p* < 0.05 vs. E169 (5 mg/kg) + MK801-treated mice.

Interestingly, the results observed for the effects of acute systemic administration of E169 versus the control mice group showed that there was no significant difference between different groups treated with E169 (2.5 and 5 mg/kg) with (F(1,10) = 0.05, *p* = 0.81) and (F(1,10) = 0.21, *p* = 0.65), respectively, nor in comparison with VEH-treated control mice (Figure 5A). However, the higher dose of E169 (10 mg/kg, i.p.) significantly decreased the DI value (F(1,10) = 7.12, *p* < 0.05) (Figure 5A). Similar to the results observed for STM, the mice group injected with LY (7.5 mg/kg, i.p.) exhibited a significant decrease in DI (F(1,10) = 9.59, *p* < 0.05) in comparison with the VEH-treated control mice, signifying the negative effect of LY on the memory of control mice (Figure 5A). Additionally, and similar to the results observed for LY on STM, mice injected with RAMH showed a significant reduction in their memory, with (F(1,10) = 27.68, *p* < 0.001) in comparison with VEH-treated control mice (Figure 5A).

Results for LY294002 are shown in Figure 5A,B. Statistical analyses showed a significant reduction in the DI value for the group pretreated with MK801 (F(1,10) = 10.01, *p* < 0.05) compared to the VEH-treated control group (Figure 5B). However, mice with MK801-induced amnesia showed a considerable enhancement in the observed DI values (F(1,10) = 11.23, *p* < 0.01) following systemic treatment with E169 (5 mg/kg, i.p.). However, amnesic mice treated with the lower (2.5 mg/kg, i.p.) or the higher (10 mg/kg, i.p.) dose of E169 showed no significant increase in the observed DI values, with (F(1,10) = 0.02, *p* = 0.89) and (F(1,10) = 1.09, *p* = 0.31) in comparison with control amnesic mice (Figure 5B), demonstrating dose-dependent effects for the tested compound E169. Moreover, amnesic mice when pretreated with LY (7.5 mg/kg, i.p.) showed a significant DI increase (F(1,10) = 7.95, *p* < 0.05) (Figure 5B), demonstrating the positive effect of LY294002 on MK801-induced amnesia in mice. Furthermore, the statistical analyses of the observed results showed that LY co-injected with E169 (5 mg/kg, i.p.) failed to further increase DI (F(1,10) = 0.09, *p* = 0.76) compared to the E169-only treated group, demonstrating no synergistic effects of combining E169 with LY on MK801-induced long-term memory deficits in mice. However, the effect observed with the latter combination was significantly higher than that of the VEH-treated control amnesic group with (F(1,10) = 16.23, *p* < 0.01) (Figure 5B). In addition, and in a further abrogative study, the CNS-penetrant H3R agonist RAMH was able to counteract the memory-enhancing effects observed with E169 (5 mg/kg, i.p.), with (F(1,10) = 5.71, *p* < 0.05) compared with the E169 (5 mg)-treated amnesic group.

### 2.4. E169 Mitigated the Level of Disturbance in Expression of PI3K, Akt, and GSK-3β Proteins in the Hippocampus of Treated Mice

**Figure 6:**
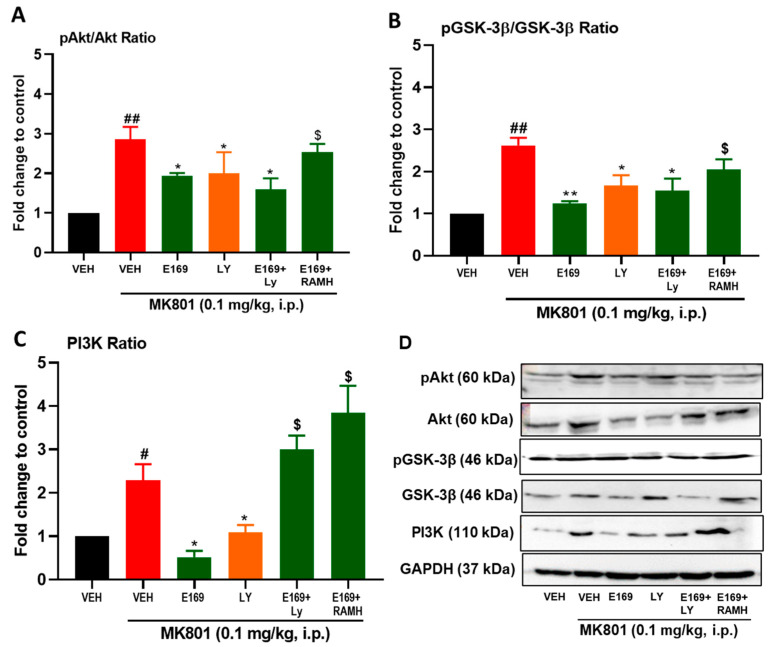
E169 mitigated the level of disturbance in the expression of PI3K, Akt, and GSK-3β proteins in the hippocampus of treated mice. (**A**) pAkt/Akt ratio, (**B**) pGSK-3β/GSK-3β ratio and (**C**) PI3K ratio was calculated using the protein expression levels of the corresponding proteins. (**D**) Immunoblotting patterns of proteins in the experiment samples. The graph represents the means ± SEM (n = 3). ^#^ *p* < 0.05 ^##^ *p* < 0.01 vs. VEH treated; * *p* < 0.05; ** *p* < 0.01 vs. VEH-treated mice with MK801-induced amnesia; ^$^ *p* < 0.05 vs. E169 (5 mg/kg, i.p.)-treated mice with MK801-induced amnesia.

The observed results showed that control mice exhibited normal hippocampal levels of PI3K, pAKT/AKT ratio, and pGSK-3β/GSK-3β ratio. However, MK801-treated mice showed significant disturbances in protein expression of the cascade witnessed as a significant increase in the expression of PI3K (F(1,5) = 8.70, *p* < 0.05), pAKT/AKT ratio (F(1,5) = 33.88, *p* < 0.01), and pGSK-3β/GSK-3β ratio (F(1,5) = 72.18, *p* < 0.01), and as compared to the hippocampal tissues of VEH-treated control mice group (Figure 6A–D).

However, animals treated with E169 (5 mg/kg, i.p.) exhibited a significant reduction in expression of the cascade proteins witnessed as a considerable reduction in protein expression of PI3K (F(1,5) = 8.31, *p* < 0.05), pAKT/AKT ratio (F(1,5) = 8.39, *p* < 0.05), and pGSK-3β/GSK-3β ratio (F(1,5) = 48.64, *p* < 0.01) in comparison with the MK801-treated amnesic mice group (Figure 6A–D). Moreover, mice injected with LY (7.5 mg/kg, i.p.) exhibited a considerable decrease in expression of proteins of the cascade as a result of a significant reduction in protein expression of PI3K (F(1,5) = 8.96, *p* < 0.05), pAKT/AKT ratio (F(1,5) = 7.95, *p* < 0.05), and pGSK-3β/GSK-3β ratio (F(1,5) = 9.02, *p* < 0.05) compared to MK801-treated amnesic mice (Figure 6A–D). Interestingly, the group that was co-injected with E169 (5 mg/kg) and LY (7.5 mg/kg) exhibited a significant reduction in expression of proteins protein of the cascade as a result of a considerable reduction in protein expression of PI3K (F(1,5) = 10.70, *p* < 0.05), pAKT/AKT ratio (F(1,5) = 8.83, *p* < 0.05), and a pGSK-3β/GSK-3β ratio (F(1,5) = 9.53, *p* < 0.05) in comparison with MK801-treated mice (Figure 6A–D). However, there were no significant effects of the combined test compounds when compared to each one tested alone. In addition, systemic co-administration of the H3R agonist RAMH (10 mg/kg) abrogated the E169 (5 mg)-provided mitigating effects on protein expression and of PI3K (F(1,5) = 9.02, *p* < 0.05), pAKT/AKT ratio (F(1,5) = 8.09, *p* < 0.05), and pGSK-3β/GSK-3β ratio (F(1,5) = 11.45, *p* < 0.05) compared to the E169 (5 mg)-treated amnesic mice group (Figure 6A–D), indicating the abrogative properties of RAMH on the effects provided by E169 (5 mg/kg).

### 2.5. Effects of E169 on Disturbed Anxiety-Like Levels and Locomotor Activity of Tested Mice

#### 2.5.1. Results for Control Mice Assessed on STM and LTM in OFT

The results observed for the effects of systemic treatments on time spent in the center, time spent in the periphery, and total distance traveled are listed in Table 1. The results observed for the reference drug LY294002 (7.5 mg/kg) revealed a considerable reduction in the amount of time spent in the center arena combined with a significant increase in the time spent in the periphery (all *p* < 0.001), and without appreciable effect on the total distance traveled. However, results showed that groups treated with E169 (2.5, 5, and 10 mg/kg, i.p.) failed to modulate time spent in the center, with (F(1,10) = 0.29, *p* = 0.60), (F(1,10) = 0.33, *p* = 0.57), and (F(1,10) = 0.02, *p* = 0.88), respectively. Additionally, the time spent in the periphery was not affected following systemic administration with E169 (2.5, 5, and 10 mg/kg, i.p.), with (F(1,10) = 2.37, *p* = 0.15), (F(1,10) = 3.72, *p* = 0.08), and (F(1,10) = 4.26, *p* = 0.06), respectively. Interestingly, the group that received RAMH showed a significant reduction in the time spent in the center (F(1,10) = 20.53, *p* < 0.01), with an increment in the amount of time spent peripherally (F(1,10) = 7.95, *p* < 0.05). Similar to the results observed on STM, the results observed in LTM revealed a considerable reduction in the amount of time spent centrally and an increase in the amount of time spent peripherally (all *p* < 0.001), and no appreciable alteration of total distance traveled following systemic administration with LY (7.5 mg/kg). Additionally, groups treated with E169 (2.5, 5, and 10 mg/kg, i.p.) did not show any appreciable alteration in the time spent in the center, with (F(1,10) = 1.80, *p* = 0.20), (F(1,10) = 2.92, *p* = 0.11), and (F(1,10) = 1.27, *p* = 0.28), respectively. Moreover, the assessment of time spent in the periphery revealed that systemic administration of E169 (2.5, 5, and 10 mg/kg, i.p.) failed to provide any significant changes, with (F(1,10) = 1.46, *p* = 0.25), (F(1,10) = 1.59, *p* = 0.23), and (F(1,10) = 2.09, *p* = 0.17), respectively. In line with the results observed on STM, mice that received RAMH in LTM showed a considerable reduction in the amount of time spent centrally and an increase in time spent in the periphery (all *p* < 0.001), without appreciable effects on the total distance traveled (Table 1).

#### 2.5.2. Results for Mice with MK801-Induced Amnesia on STM and LTM in OFT (Amnesic Mice)

The results observed revealed that amnesic mice on STM showed a considerable reduction in the amount of time spent in the center and an increase in time spent in the periphery (all *p* < 0.0001), but no significant effect was observed on the total distance traveled. LY (7.5 mg/kg, i.p.) showed a considerable increment in the amount of time spent centrally and an increase in time spent in the periphery of amnesic mice (all *p* < 0.05). Additionally, E169 (2.5 mg/kg, i.p.) had no significant effects on the time spent in the center or periphery, with (F(1,10) = 1.82, *p* = 0.20) and (F(1,10) = 0.004, *p* = 0.94), respectively. However, amnesic mice that received E169 (5 or 10 mg/kg, i.p.) showed a significant increase in the time spent in the center (F(1,10) = 14.59, *p* < 0.01) and (F(1,10) = 7.96, *p* < 0.05), and periphery (F(1,10) = 6.32, *p* < 0.05) and (F(1,10) = 5.00, *p* < 0.05), respectively, without any appreciable effect on the total distance traveled. Moreover, systemic co-administration of amnesic mice with RAMH (10 mg/kg, i.p.) and E169 (5 mg/kg, i.p.) showed a significant reduction in time spent in the center and an increase in time spent in the periphery (all *p* < 0.05) in comparison with mice treated only with E169 (5 mg/kg), without any effects observed on the measured total distance traveled. In an additional experiment, amnesic mice treated with a combination of LY (7.5 mg/kg) and E169 (5 mg/kg) showed a considerable increase in the amount of time spent centrally, accompanied by a significant reduction in the time spent peripherally (all *p* < 0.05), with no significant difference observed when compared to the amnesic mice group treated with E169 (5 mg/kg) only. Similarly, the results observed for amnesic mice in LTM showed a considerable reduction in the time spent centrally with an increase in the time spent peripherally (all *p* < 0.05), and without any effect on total distance traveled. In addition, the results revealed that amnesic mice injected with LY294002 (7.5 mg/kg, i.p.) showed a significant increment in the time spent in the center and a significant reduction in the time spent in the periphery (all *p* < 0.05). Groups pretreated with E169 (2.5 or 10 mg/kg, i.p.) failed to show any significant change in the amount of time spent centrally (F(1,10) = 1.30, *p* = 0.27) and (F(1,10) = 3.13, *p* = 0.10), or time spent in the periphery (F(1,10) = 0.88, *p* = 0.30), (F(1,10) = 0.03, *p* = 0.85). However, amnesic mice treated with E169 (5 mg/kg, i.p.) showed a significant increase in time spent centrally and a significant reduction in time spent peripherally (all *p* < 0.05), with no effect on total traveled distance. Moreover, co-administration of RAMH reversed the effects observed with E169 (5 mg/kg) on the amount of time spent centrally and time spent peripherally (all *p* < 0.05), without any significant alteration of total distance traveled. Furthermore, amnesic mice co-injected with LY294002 (7.5 mg/kg) and E169 (5 mg/kg) showed a significant increase in time spent in the center (F(1,10) = 5.68, *p* < 0.05) and a significant reduction in the amount of time spent peripherally (F(1,10) = 7.61, *p* < 0.05) in comparison with VEH-treated amnesic mice, and compared to amnesic mice treated with E169 (5 mg/kg) only (Table 1).

## 3. Discussion

The role of the brain’s histaminergic system in AD has been proposed, and a variety of H3R antagonists/inverse agonists targeting central histaminergic systems, and specifically H3Rs, have been developed. However, no previous study investigated whether the memory-enhancing effects of H3R antagonists are also due to any simultaneous mitigating effects of the respective H3R antagonist on the expression of proteins in the Akt-mTOR signaling pathway in specific brain regions, e.g., hippocampus, of tested animals. Therefore, in the current project the effects of the newly developed highly potent and selective non-imidazole H3R antagonist, E169 [1-(6-(naphthalen-1-yloxy)hexyl)azepane], with high affinity [16] were evaluated on MK801-induced memory deficits, applying novel object recognition tests (NORT) and assessing treated mice on short- and long-term memory (STM and LTM). In addition, the simultaneous modulating effects of E169 on the level of disturbance in the expression of PI3K, Akt, and GSK-3β proteins were assessed in the hippocampus of treated mice with MK801-induced amnesia, to clarify whether H3R antagonism has a simultaneous role in the Akt-mTOR signaling pathway in the hippocampal tissues of treated animals. Additionally, since the effects of test compound E169 may affect anxiety and motor activity, and, therefore, could confound the learning and memory performance of tested animals [19], the effects of E169 and the reference PI3K inhibitor LY294002 on locomotor activity and anxiety-like behaviors of the same animals following NORT were investigated in OFT. Moreover, the abrogative effects of the H3R agonist RAMH on the E169-provided memory-enhancing effects in NORT were evaluated.

Our results show that the H3R antagonist E169 effectively counteracted the deteriorating effects induced by systemic injection of MK801 on STM (Figure 4B) and LTM (Figure 5B), since E169 improved the performance of tested mice in the NORT for both STM and LTM by increasing the observed DI values, indicating the memory-improving properties of the H3R antagonist E169. Our observations agree with previous results obtained for the H3R antagonist/inverse agonist ABT-239 in the same test model [20]. Moreover, our results are in agreement with several previous preclinical observations applying NORT in rats and mice and assessing numerous H3R antagonists, e.g., thioperamide and clobenpropit [21]; pitolisant [22]; GSK189254; SAR110894 [23,24]. All of them were found to enhance induced memory deficits following systemic injection of scopolamine and/or MK801. Moreover, our observed results show that the E169-provided memory-enhancing effects on MK801-induced amnesia on STM and LTM are comparable to the standard PI3K inhibitor LY294002 (LY) and to the reference drug donepezil (DOZ, acetylcholinesterase inhibitor). The H3Rs functions as an H3 auto-receptor modulating synthesis and release of central HA [23,25], and as an H3 hetero-receptor located on neurons other than histaminergic neural cells and moderating the brain levels of several other neurotransmitters, e.g., ACh, DA, 5-HT, gamma-aminobutyric acid (GABA), and Glu that are involved in memory function [7,8,26]. Therefore, the effects observed due to E169 could be due to its capability as an H3R antagonist to increase the brain level of histamine (H3 auto-receptor function) and other memory-enhancing neurotransmitters including ACh (H3 hetero-receptor function). Interestingly, numerous H3R antagonists/inverse agonists were found to exhibit a unique feature by their cognition-enhancing properties as indicated by several lines of evidence from preclinical studies in different rodents. Notably, the combined systemic administration of the most promising dose of the test compound E169 (5 mg/kg) together with the PI3K inhibitor LY (7.5 mg/kg) failed to provide any synergistic effect when compared to E169 or LY administered alone. The failure to obtain any synergistic effects of the combined treatment might be explained by the different mechanisms of both compounds and by the ability of LY to deteriorate memory performance in control mice (STM, Figure 4B and LTM, Figure 5B). Interestingly, the E169-provided benefits in NORT were completely reversed when mice were concurrently injected with the centrally acting H3R agonist RAMH (Figure 4B and Figure 5B). The latter findings are consistent with a previous study in which RAMH nullified the memory-improving effects of the H3R antagonist ciproxifan on LTM in experimental rodents [27]. 

Furthermore, another goal of the current project was to examine whether E169 modulated the PI3K-AKT-GSK3β signaling pathway and therefore participated in the observed memory-enhancing effects in amnesic mice. Western blot analyses of our observed results indicated that acute systemic treatment with the H3R antagonist E169 resulted in a significant reduction in the cascade-protein expression, as witnessed as a significant reduction in the protein expression of PI3K, pAKT/AKT ratio, and pGSK-3β/GSK-3β ratio compared to the protein levels of hippocampal tissues of amnesic control mice (VEH) (Figure 6). The latter results indicated that the test compound E169 improved memory deficits induced by MK801, since the recognition memory of MK801-treated mice with continuously active GSK-3β, resulting in low levels of pGSK-3β at the Ser-9 residue, was diminished. The latter observations are in agreement with previous preclinical experiments in which impairment of recognition memory was seen in transgenic mice with continuously active GSK-3β and low levels of pGSK-3β at Ser9 [28]. In a further experiment, mice which were co-administered with the CNS-penetrant H3R agonist RAMH, failed to display these beneficial effects of E169 on phosphorylation of the GSK-3β, and this agrees with previous results observed in preclinical experiments in rodents [29] and suggests that the integrity of the brain’s histaminergic neurotransmission system is required for the behavioral and biochemical effects triggered by H3R antagonists, e.g., E169, and also by H3R agonists, e.g., RAMH. Our observations also showed that the phosphorylation of Ser473-Akt, and Ser9-GSK-3β in the hippocampus of treated mice was overactivated by the amnesic effects induced by MK801, and these results agreed with previous preclinical experiments that were carried out in experimental rodents [30]. Notably, it has been suggested that H3R antagonists can enhance cognitive functions in a variety of cognitive disorders by increasing the release of ACh and inducing pGSK-3β [31,32], which is comprehended with our test compound E169 functioning also as an H3 hetero-receptor antagonist

In the OFT, our observations showed that locomotion and anxiety levels at administered doses were not affected, as the observed time spent in the periphery, the time spent in the center, and the total distance traveled for mice pretreated with E169 (2.5–10 mg/kg) did not differ from the control group. Consequently, the observed results in OFT excluded all these confounding factors. These results in OFT are, also, in agreement with recent previous observations in which acute systemic administration of several H3R antagonists, e.g., DL77 (2.5–10 mg/kg) and E159 (2.5–10 mg/kg) failed to modify spontaneous locomotor activity of the same animal species in the OFT [6]. These results are of high significance since improved performance in NORT can be the consequence of several variables not related to memory-enhancing effects of test compound E169, such as modifications in emotional response or in spontaneous locomotor activity [33]. Therefore, our observations showed that the H3R antagonist E169 was capable of counteracting cognitive deficits by simultaneously modulating barin HA and several other brain neurotransmitters by functioning as antagonist on H3 auto- and hetero-receptors and by mitigating disturbances in hippocampal protein levels in the PI3K/AKT/GSK-3β signaling pathway in the context of MK801-induced amnesia in mice, without any modulating effects on locomotor activity or anxiety-like behaviors of tested animals.

## 4. Materials and Methods

### 4.1. In Silico Calculations

#### 4.1.1. ADMET Calculations

Physicochemical and drug-likeness properties of E169 were calculated using the server SwissADME [http://www.swissadme.ch (Last accessed on 4 January 2023)].

Pharmacokinetic properties of E169 were calculated using the server SwissADME [http://www.swissadme.ch (Last accessed on 4 January 2023)] and the server pkCSM [https://biosig.lab.uq.edu.au/pkcsm (Last accessed on 4 January 2023)].

Toxicity properties of E169 were calculated using the server pkCSM [https://biosig.lab.uq.edu.au/pkcsm (Last accessed on 4 January 2023)] and the server ProToxII [https://tox-new.charite.de (Last accessed on 4 January 2023)].

Phase I transformation of E169 was predicted using the server Biotransformer [http://biotransformer.ca/new (Last accessed on 4 January 2023)].

#### 4.1.2. Molecular Docking Studies

For docking purposes, Schrodinger 2022-4 was used. Ligands were built in their ionized forms (protonated N4 piperazine nitrogen, structure charge +1). Bioactive conformations were generated using ConfGen [34] (water environment, target number of conformers—20). For all the compounds, the 5 lowest energy conformers were selected for docking studies. Docking to the rigid form of the receptor (bound ligand-centered grid) was performed using a standard docking protocol [35]. To validate the methods used, the native ligand PF03654746 was redocked with high confidence. Complex energy of the docked pose was calculated using Prime MM-GBSA Dynamics simulations (in time of 100 ns, T = 300 K) which were run in Desmond [36]. The protein orientation in the membrane was obtained from the OPM database [37]. Simulations lasted for 100 ns and the TIP3P solvent model was applied [38]. A total of 1000 frames were produced for the run.

### 4.2. Animals

C57BL/6 male mice (aged 8–12 weeks, weighing 20–25 g) were acquired from the animal facility at the College of Medicine and Health Sciences at the United Arab Emirates University. The animals were kept in a separate air-conditioned room with a 12 h light/dark cycle and a controlled temperature and humidity (24 ± 2 °C and 55 ± 15%, respectively). All the experiments took place between 8 a.m. and 2 p.m., male mice were used to decrease within-group variability due to hormonal fluctuations during an estrogen cycle in female mice. The procedures used to assess the effects of H3R antagonists were approved by the Institutional Animal Ethics Committee of CMHS/UAEU (ERA-2019_6013). All possible efforts were undertaken to reduce the number of animals used and their suffering. Additionally, all behavioral studies were conducted by the same experimenter.

### 4.3. Drugs

The test compound E169 was synthesized and pharmacologically profiled in the Department of Technology and Biotechnology of Drugs (Jagiellonian University Medical College, Kraków, Poland) as previously described [16] (Figure 1). The PI3K inhibitor LY294002 (LY) was purchased from EMD Millipore. The CNS-penetrant MK801 hydrogen maleate (dizocilpine) and the H3R agonist (R)-α-methylhistamine (RAMH) were purchased from Sigma-Aldrich. All drugs were dissolved in physiological saline containing 1% dimethyl sulfoxide (DMSO). A volume of 12 mL/kg of body weight was used to standardize the volume, and each dose of tested compounds was described in terms of the free base.

### 4.4. Reagents and Antibodies

The blocking agent bovine serum albumin (BSA) was purchased from Sigma-Aldrich. The primary mouse monoclonal antibodies anti-AKT (protein kinase B) (PKB), anti-pAKT (phosphorylated protein kinase B) (PKB), anti-PI3K (phosphoinositide 3-kinase), anti-GSK-3β (glycogen synthase kinase-3), anti-pGSK-3β (phosphorylated glycogen synthase kinase-3), and anti-GAPDH (glyceraldehyde-3-phosphate dehydrogenase), as well as the secondary antibody (anti-mouse polyclonal IgG), were purchased from Santa Cruz Biotechnology. The blotting polyvinylidene fluoride (PVDF) membrane was acquired from Bio-Rad Laboratories (Hercules, CA, USA). Thermo Fisher Scientific (Rockford, IL, USA) was the source of the chemiluminescence pico-kit and the PierceTM BCA protein assay kit. 

### 4.5. Behavioral Tests

#### 4.5.1. Novel Object Recognition Test (NORT)

This test setup included an “open-field” arena (45 × 45 × 30 cm) in a sound-attenuated room. Additionally, three objects (cubes) were used: two of them were identical in shape and the other had a distinct shape following a previous protocol [39]. The NORT consisted of three phases: habituation phase, training session (T1), and test session (T2). All mice were acclimated to the experimental apparatus for 10 min (habituation) in the absence of objects 24 h before training. During the training session (T1), mice were re-introduced to the arena in the presence of two identical plastic objects (cubes) and observed for 5 min. The test session (T2) was carried either 2 h after T1 to assess STM in one set of animals, or 24 h after T1 to investigate LTM in a different set of animal groups (Figure 4 and Figure 5).

During the test session (T2), each mouse was left in the testing arena for 5 min with one of the old objects and another new object and observed for 5 min. The arena and test objects were meticulously cleaned with 70% (*v*/*v*) ethanol between trials to eliminate any olfactory or/and taste signs. During the training and testing sessions the time spent actively exploring the objects was recorded and scored by using a charge-coupled device with a camera-assisted motion-tracking apparatus and software (EthoVision 3.1, Noldus Information Technology, Wageningen, The Netherlands). Exploration included using the nose and/or forepaws to sniff or touch the objects. To prevent bias from a preference for order or location, the object positions (left/right) were randomly chosen.

The discrimination index was calculated using this formula:

DI = (T2 − T1)/(T2 + T1) × 100

T2 represents time spent exploring the novel object. 

T1 represents time spent exploring the familiar object.

The administration of drugs was as follows:

- MK801 (0.1 mg/kg i.p.) was injected 15 min before (T1) and (T2) sessions;
- E169 (2.5–10 mg/kg i.p.) was injected 60 min before (T1) and (T2) sessions;
- LY294002 (LY; 7.5 mg/kg, i.p.) was injected 75 min before (T1) and (T2) sessions;
- RAMH (10 mg/kg, i.p.) was injected 30 min before (T1) and (T2) sessions;
- VEH (DMSO) was injected at all these time points, 75, 60, 30, 15 min, before (T1) and (T2).

The doses of the test compounds were as follows: MK801 (0.1 mg/kg), E169 (2.5–10 mg/kg), LY294002 (7.5 mg/kg), RAMH (10 mg/kg). The vehicle (VEH, DMSO) was injected to remove any biased effect of the injection procedure or the vehicle itself on memory. As mentioned in the introduction, MK801 induces amnesia by blocking the NMDA receptors. Accordingly, MK801-injected mice represent a model of impaired memory compared to the control group injected with the VEH. Furthermore, pretreatments with different compounds were carried out to assess their effects on memory in both groups, the group in which amnesia was induced following systemic administration of MK801, and the control VEH-treated group.

#### 4.5.2. Open Field Test (OFT)

The OFT was used to detect the effect of the treatments (E169, MK801, RAMH, and LY) on anxiety-like behaviors as well as locomotor activity of tested animals [19,40,41,42,43]. The OF box was a square box with dimensions of 45 × 45 × 30 cm, the central arena was defined as a 23 cm by 23 cm square in the center zone, the remainder designated as the peripheral area. Briefly, the test included acclimatization for 5 min to familiarize the mice with the environment, then recording their activity for 5 min using a charge-coupled device with a camera-assisted motion tracking system and software (EthoVision 3.1, Noldus Information Technology, The Netherlands). The total distance traveled in the entire arena, as well as time spent in the center and the peripheral zone, were recorded. Following each mouse’s completion of the test, the open field-testing area was carefully wiped with 70% ethanol. Usually, excessive anxiousness causes experimental mice to spend less time in the center of the arena and more time in closer to the walls (in the periphery). On the other side, a longer time spent in the center of the test arena suggests lower anxiousness and more exploratory behaviors of tested animals [44].

### 4.6. Biochemical Assessment

#### 4.6.1. Brain Collection and Tissue Processing

Following a previously reported protocol [45,46], the animals were sacrificed at the end of the behavioral tests. Pentobarbital was used to tranquilize the animals (40 mg/kg body weight, i.p.) and then blood was washed out with phosphate buffer solution, PBS 1× (0.01 M phosphate buffer, 0.0027 M potassium chloride, and 0.137 M sodium chloride), at pH 7.4 by intracardial infusion. The evacuation of blood was confirmed by the pale hue of the liver, heart, and kidney, confirming that they were blood-free. After being excised from the brain, the hippocampus was deposited in liquid nitrogen and kept at −80 °C for later testing [47]. The tissues were homogenized in a RIPA buffer containing protease inhibitors and phosphatase inhibitors to prevent protein degradation and the homogenates were centrifuged at 16,000 rpm/min for 20 min at 4 °C to remove tissue debris. After centrifugation, the supernatants were collected and stored at −20 °C and used later to determine the levels of PI3K, AKT, pAKT, GSK-3β, and pGSK-3β using western blot. The PierceTM BCA protein assay kit was used to determine the protein concentrations in each protein sample (Thermo Table Fisher Scientific, Rockford, IL, USA).

#### 4.6.2. Western Blotting

According to our results in protein estimation, we chose a certain volume of the samples for loading the gel, the protein samples were separated on 12% SDS–polyacrylamide gels and transferred to PVDF membranes that had previously been activated with 100% methanol using two machines for running the gel and transferring the gel, both purchased from the BIO-RAD company. After that, the membranes were incubated for one hour with 5% bovine serum albumin (BSA) at 4 °C to block the non-protein background of the membrane, then the membranes were incubated at 4 °C with gentle rocking overnight with specific primary antibodies against GAPDH, PI3K, AKT, pAKT, GSK-3β, and pGSK-3β (1:1000). Before the membranes were incubated with a secondary antibody for one hour at 4 °C, they were thoroughly rinsed with three cycles of tris-buffered saline (TBS), each cycle for 5 min. After the secondary antibody, the membranes were once again thoroughly rinsed with tris-buffered saline (TBS) for three cycles, and the protein bands were detected using a Thermofisher pico kit with enhanced chemiluminescence. ImageJ software was used to measure the band intensity for determining protein levels (NIH, USA).

### 4.7. Statistical Analyses

Data for behavioral tests were given as means with standard error of the mean (SEM), compound effects were explored using two-way analysis of variance (ANOVA), and post hoc comparisons were performed using Tukey’s test if a significant main effect was discovered. Data from western blots were analyzed using one-way ANOVA, and then a post hoc Tukey’s multiple comparison test was performed. The statistical comparisons were performed using the Prism GraphPad program. *p* values below 0.05 (*p* < 0.05) were used to denote statistical significance.

## 5. Conclusions

De Almeida and Izquierdo made the initial suggestion on the function of the brain’s HA in the regulation of various memory phases in 1986. There is strong evidence linking changes in the brain’s histaminergic neurotransmission system to the cognitive deficits witnessed in several neurodegenerative disorders. The current research work demonstrates that the highly potent and selective H3R antagonist/inverse agonist E169 and the PI3K inhibitor LY294002 showed memory-enhancing effects on MK801-induced amnesia in tested male adult mice. The observed effects could be attributed to the capability of the H3R antagonist E169 to modulate brain histaminergic neurotransmission, as the CNS-penetrant H3R agonist RAMH reversed the E169-provided effects on the memory performance and the levels of protein expression assessed in the tested mice. Moreover, the observed effects clearly demonstrated that E169 mitigated the disturbance in the PI3K/AKT/GSK-3β signaling pathway through its selective H3R antagonistic effect, therefore balancing the disturbed phosphorylation of GSK-3β caused following amnesia induced by MK801.

Therefore, the results observed in the current series of experiments clearly showed that H3R antagonism counteracted cognitive deficits by simultaneously modulating brain levels of HA and several other neurotransmitters by functioning as an H3R antagonist on H3 auto- and hetero-receptors, respectively, and by mitigating disturbances in hippocampal protein levels in the PI3K/AKT/GSK-3β signaling pathway following MK801-induced amnesia in mice. These obtained results suggest the potential of H3R antagonists such as E169, with good in silico physicochemical properties and stable retained key interactions in docking studies at H3R, to simultaneously modulate disturbances in brain neurotransmitters and imbalanced in the Akt-mTOR signaling pathway related to neurodegenerative disorders, e.g., AD.

## 6. Study Limitations and Future Directions

With the observed results in the current series of experiments and to enable a conclusive correlation between the effects observed and histaminergic neurotransmission through H3Rs, additional experiments are still warranted to explore whether the H3R antagonist E169 and agents promoting PKI3 activity counteract or complement each other’s effects on the PI3K/AKT/GSK-3 signaling pathway. Additionally, further experiments are required to assess whether the CNS-penetrant RAMH is capable of counteracting the effects provided by the PI3K inhibitor LY294002. Further behavioral assessments using E169 and other H3R antagonists are needed in a wide range of other animal models with AD to be able to generalize our current findings. This will provide sufficient pharmacological evidence with the aim to suggest a prospective related therapeutic approach in AD interventions.

## Figures and Tables

**Table 1:** E169 altered disturbed anxiety-like levels without affecting locomotor activity in tested mice.

Group	Time Spent in Center (s)		Time Spent in Periphery (s)		Total Distance Travelled (cm)	
A. Control	STM	LTM	STM	LTM	STM	LTM
VEH	123.86 ± 0.37	156.91 ± 4.54	176.14 ± 1.41	143.09 ± 18.62	3304.84 ± 206.76	2884.67 ± 172.10
E169 (2.5 mg)	118.35 ± 5.58	152.59 ± 4.20	181.65 ± 11.26	147.41 ± 7.50	3140.35 ± 257.18	2670 ± 47.31
E169 (5 mg)	146.51 ± 5.45	149.71 ± 7.50	153.49 ± 11.64	150.29 ± 10.87	3093.34 ± 215.06	2487.24 ± 110.40
E169 (10 mg)	150.30 ± 7.78	159.21 ± 10.08	149.70 ± 12.72	140.79 ± 4.41	3145.25 ± 165.51	2570.80 ± 83.06
LY294002	75.22 ± 7.44 ***	109.27 ± 6.48 ***	224.78 ± 9.33 ***	190.73 ± 10.41 **	3106.14 ± 180.92	2745.22 ± 145.24
RAMH	90.22 ± 10.65 **	112.56 ± 7.68 ***	209.78 ± 11.80 *	187.44 ± 14.90 *	3002.52 ± 309.43	2686.43 ± 81.74
**B. MK801**	**STM**	**LTM**	**STM**	**LTM**	**STM**	**LTM**
VEH	97.70 ± 3.03 ^####^	121.12 ± 6.07 ^##^	202.3 ± 10.98 ^#^	178.88 ± 4.56 ^#^	2701 ± 285.58	2531.66 ± 42.18
E169 (2.5 mg)	110.95 ± 9.33	131.47 ± 6.64	189.05 ± 16.68	168.53 ± 9.564	2854.33 ± 71.75	2648.32 ± 104.28
E169 (5 mg)	135.54 ± 9.42 **	154.08 ± 6.63 *	164.46 ± 6.33 *	145.92 ± 10.67 *	2801.36 ± 91.97	2705.98 ± 90.67
E169 (10 mg)	122.98 ± 8.42 *	133.45 ± 5.75	177.02 ± 5.77 *	166.55 ± 11.73	2794.12 ± 65.99	2629.48 ± 60.76
LY294002	138.58 ± 5.36 ****	148.29 ± 6.58 *	161.42 ± 6.84 *	151.71 ± 4.44 *	2956.03 ± 37.58	2722.14 ± 133.25
E169 + RAMH	111.49 ± 4.74 ^$^	118.18 ± 2.36 ^$^	188.51 ± 4.24 ^$$^	181.82 ± 6.55 ^$^	2807.93 ± 93.09	2744.54 ± 81.64
E169 + LY294002	152.52 ± 8.80 *	148.62 ± 8.27 *	147.48 ± 10.02 *	151.38 ± 12.79 *	2797.74 ± 105.52	2700.49 ± 104.72

The table represents the means ± SEM (n = 6). (A) * *p* < 0.05; ** *p* < 0.01; *** *p* < 0.001 vs. VEH-treated control mice; (B) ^#^ *p* < 0.05; ^##^ *p* < 0.01 ^####^ *p* < 0.0001 vs. VEH-treated control mice; * *p* < 0.05; ** *p* < 0.01; **** *p* < 0.0001 vs. MK801-treated group; ^$^ *p* < 0.05; ^$$^ *p* < 0.01 vs. E169 (5 mg/kg, i.p.) + MK801-treated group.

## Data Availability

Data are contained within the article.

## Supplementary Materials

The following supporting information can be downloaded at: https://www.mdpi.com/article/10.3390/ijms241612719/s1, Results of performed calculations: Table S1, Physicochemical properties of E169 calculated by SwissADME; Table S2, Drug-likeness properties of E169 calculated by server SwissADME; Table S3, Pharmacokinetic properties of E169 calculated by server SwissADME and pkCSM; Table S4, Toxicity properties of E169 calculated by server pkCSM; Table S5, Toxicity properties of E169 calculated by server ProToxII; Table S6, Metabolism of E169 predicted by server Biotransformer.
