# Supplementary material for: The Potent and Selective Histamine H3 Receptor Antagonist E169 Counteracts Cognitive Deficits and Mitigates Disturbances in the PI3K/AKT/GSK-3β Signaling Pathway in MK801-Induced Amnesia in Mice
Source: Int J Mol Sci. 2023 Aug 12;24(16):12719. doi: 10.3390/ijms241612719 (PMC10454630; doi:10.3390/ijms241612719)
Supplement: Supplementary file 1 [file ijms-24-12719-s001.zip › ijms-2428918-supplementary.pdf]

Supplementary data

Table S1. Physicochemical properties of **E169** calculated by server SwissADME [http://www.swissadme.ch/ (Last accessed on 4 January 2023)].

MW[a]	NHA[b]	TPSA[c]	NRotB[d]	NHBA[e]	NHBD[f]	Log Pcon[g]
325	24	12.47	8	2	0	5.21

[a]MW = molecular weight; [b]NHA = number of heavy atoms; [c]TPSA = topological polar surface area; [d]number of rotatable bonds; [e]NHBA = number of hydrogen bond acceptors; [f]NHBD = number of hydrogen bond donors; [g]log Pcon = consensus lipophilicity

Table S2. Drug-likeness properties of **E169** calculated by server SwissADME [http://www.swissadme.ch/ (Last accessed on 4 January 2023)].

Lipinski	Ghose	Veber	Egan	Muegge	PAINS
Y[a]	Y	Y	Y	N[b]	N

[a]Y= yes; [b]N = No

Table S3. Pharmacokinetic properties of **E169** calculated by server SwissADME [http://www.swissadme.ch/ (Last accessed on 4 January 2023)] and pkCSM [http://tox.charite.de/protox_II/ (Last accessed on 4 January 2023)].

**server**	BBB	GI	Substrate		Inhibitor				
			CYP2D6	CYP3A4	CYP1A2	CYP2C19	CYP2C9	CYP2D6	CYP3A4
**SwissADME**	Y[a]	Y	--[b]	--	Y	N[c]	N	Y	N
**pkSCM**	Y	Y	N	Y	Y	N	N	Y	N

[a]Y= yes; [b]-- = not predict [c]N = No

Table S4. Toxicity properties of **E169** calculated by server pkCSM [http://tox.charite.de/protox_II/ (Last accessed on 4 January 2023)].

toxicity	AMES test	hERG I inihibitor	hERG I inihibitor	hepatotoxicity
**E169**	Y[a]	N[b]	Y	N

[a]Y= yes; [b]N = No

Table S5. Toxicity properties of **E169** calculated by server ProToxII [https://biosig.lab.uq.edu.au/pkcsm (Last accessed on 4 January 2023)].

class of toxicity	predicted value of toxic dose LD_{50} [mg/kg]	predicted kind of toxicity
III[a]	178	immunotoxicity

[a] Class III: harmful if swallowed ($50 < LD_{50} \leq 300$)

Table S6. Metabolism of **E169** predicted by server Biotransformer [https://biotransformer.ca/new (Last accessed on 4 January 2023)]. Phase I transformation.

**Results ID**	Predicted results	Smiles	Chemical formula	Isotope mass [Da]	Reaction type	Reaction info	Biotransformation reaction
**1**	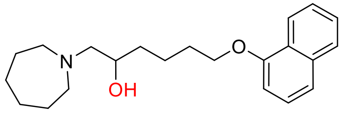	OC(CCCCOC1=CC=CC2=CC=CC=C12)CN3CCCCCC3	C22H31NO2	341,2354	Hydroxylation of acylic aliphatic secondary carbon	Enzyme: cytochrome P450 1A2 Biosystem: HUMAN	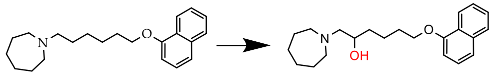
**2**	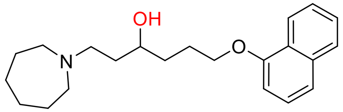	OC(CCCOC1=CC=CC2=CC=CC=C12)CCN3CCCCCC3	C22H31NO2	341,2354	Hydroxylation of acylic aliphatic secondary carbon	Enzyme: cytochrome P450 1A2 Biosystem: HUMAN	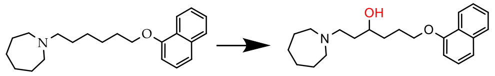
**3**	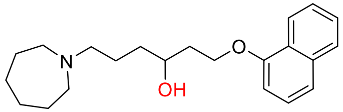	OC(CCOC1=CC=CC2=CC=CC=C12)CCCN3CCCCCC3	C22H31NO2	341,2354	Hydroxylation of acylic aliphatic secondary carbon	Enzyme: cytochrome P450 1A2 Biosystem: HUMAN	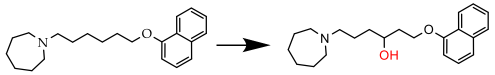
**4**	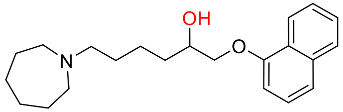	OC(COC1=CC=CC2=CC=CC=C12)CCCCN3CCCCCC3	C22H31NO2	341,2354	Hydroxylation of acylic aliphatic secondary carbon	Enzyme: cytochrome P450 1A2 Biosystem: HUMAN	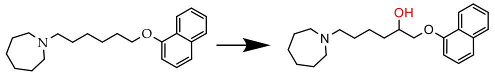

**5**	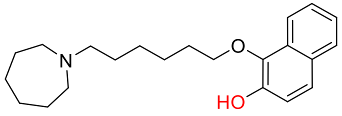	OC(C=CC1=CC=CC=C21)=C2OCCCCCCN3CCCCCC3	C22H31NO2	341,2354	Aromatic Hydroxylation of fused benzene ring	Enzyme: cytochrome P450 1A2 Biosystem: HUMAN	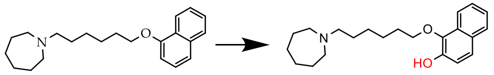
**6**	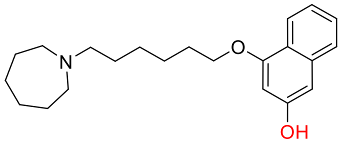	OC1=CC2=CC=CC=C2C(OCCCCCCN3CCCCCC3)=C1	C22H31NO2	341,2354	Aromatic Hydroxylation of fused benzene ring	Enzyme: cytochrome P450 1A2 Biosystem: HUMAN	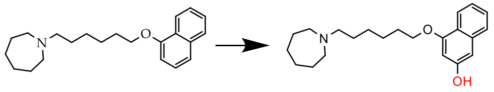
**7**	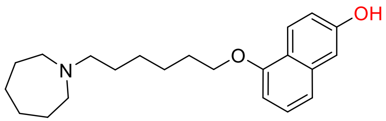	OC1=CC=C2C(OCCCCCCN3CCCCCC3)=CC=CC2=C1	C22H31NO2	341,2354	Aromatic Hydroxylation of fused benzene ring	Enzyme: cytochrome P450 1A2 Biosystem: HUMAN	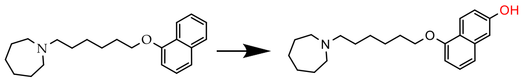
**8**	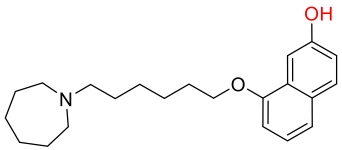	OC1=CC=C2C=CC=C(C2=C1)OCCCCCCN3CCCCCC3	C22H31NO2	341,2354	Aromatic Hydroxylation of fused benzene ring	Enzyme: cytochrome P450 1A2 Biosystem: HUMAN	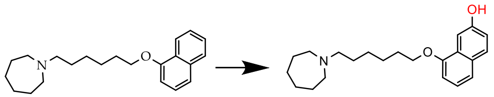
**9**	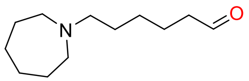	[o]=CCCCCCN1CCCCCC1	C12H23NO	197,1779	O-Dealkylation	Enzyme: cytochrome P450 1A2 Biosystem: HUMAN	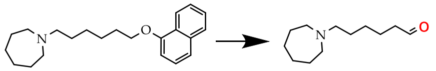

**10**	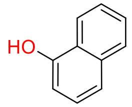	OC1=CC=CC2=CC=CC=C12	C10H80	144.0575	O-Dealkylation	Enzyme: cytochrome P450 1A2 Biosystem: HUMAN	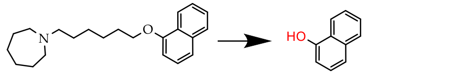
**11**	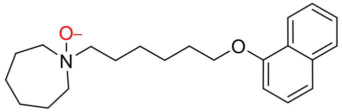	[O_][N]1(CCCCCC1)CCCCCCO C2=CC=CC3=CC=CC=C23	C22H31NO2	341.2354	N-Oxidation of alicyclic tertiary amine	Enzyme: cytochrome P450 1A2 Biosystem: HUMAN	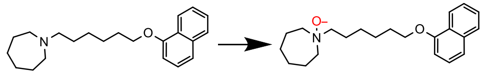
**12**	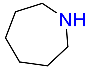	N1CCCCCC1	C6H13N	99.10479	N-Dealkylation of alicyclic tertiary amine	Enzyme: cytochrome P450 1A2 Biosystem: HUMAN	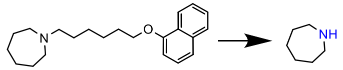
**13**	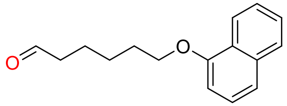	O=CCCCCCOC1=CC=CC2 =CC=CC=C12	C16H18O2	242.1306	N-Dealkylation of alicyclic tertiary amine	Enzyme: cytochrome P450 1A2 Biosystem: HUMAN	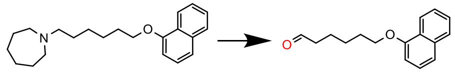
**14**	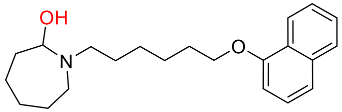	OC1N(CCCCC1)CCCCCCOC2= CC=CC3=CC=CC=C23	C22H31NO2	341.2354	Hydroxylation of heteroalicyclic secondary carbon	Enzyme: cytochrome P450 1A2 Biosystem: HUMAN	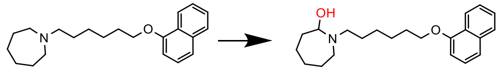

**15**	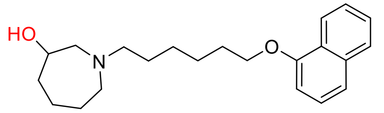	OC1CN(CCCC1)CCCCCCOC2=CC=CC3=CC=CC=C23	C22H31NO2	341.2354	Hydroxylation of heteroalicyclic secondary carbon	Enzyme: cytochrome P450 1A2 Biosystem: HUMAN	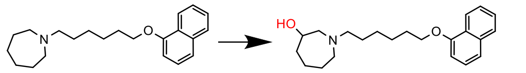
**16**	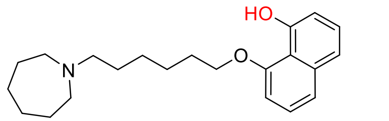	OC1=C2C(OCCCCCCN3CCCCCC3)=CC=CC2=CC=C1	C22H31NO2	341,23548	Aromatic Hydroxylation of fused benzene ring	Enzyme: cytochrome P450 1A2 Biosystem: HUMAN	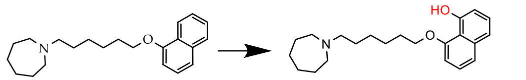
**17**	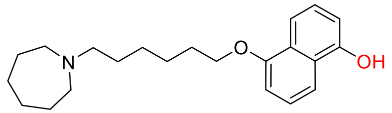	OC1=C2C=CC=C(C2=CC=C1)OCCCCCCN3CCCCCC3	C22H31NO2	341,23548	Aromatic Hydroxylation of fused benzene ring	Enzyme: cytochrome P450 1A2 Biosystem: HUMAN	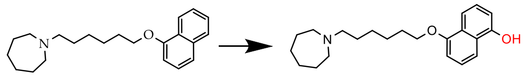
**18**	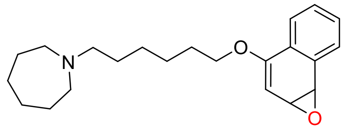	N1(CCCCCC1)CCCCCCOC2=CC([o]3)C3C4=CC=CC=C24	C22H31NO2	341.2354	Epoxidation of arene	Enzyme: cytochrome P450 1A2 Biosystem: HUMAN	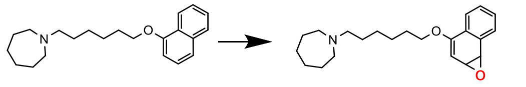
**19**	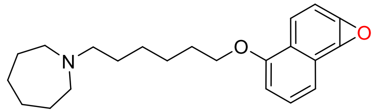	N1(CCCCCC1)CCCCCCOC2=CC=CC3=C(O4)C4=CC=C23	C22H29NO2	339.2198	Epoxidation of arene	Enzyme: cytochrome P450 1A2 Biosystem: HUMAN	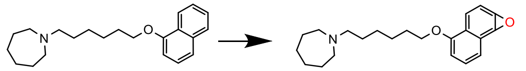

**20**	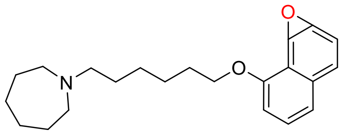	N1(CCCCCC1)CCCCCCOC2 =CC=CC3=CC=C(O4)C4=C23	C22H29NO2	339.2198	Epoxidation of arene	Enzyme: cytochrome P450 1A2 Biosystem: HUMAN	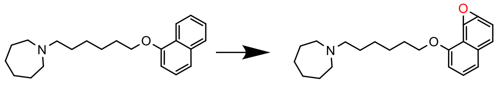
**21**	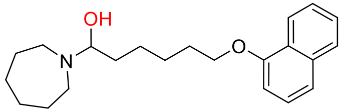	OC(CCCCCOC1=CC=CC2= CC=CC=C12)N3CCCCCC3	C22H31NO2	341.2354	Aliphatic Hydroxylation of carbon alpha to secondary or tertiary alkyl-N	Enzyme: cytochrome P450 2B6 Biosystem: HUMAN	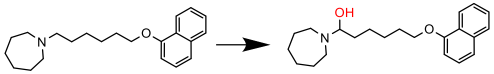
**22**	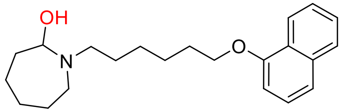	OC1N(CCCCC1)CCCCCCOC2= CC=CC3=CC=CC=C23	C22H31NO2	341.2354	Aliphatic Hydroxylation of carbon alpha to secondary or tertiary alkyl-N	Enzyme: cytochrome P450 2B6 Biosystem: HUMAN	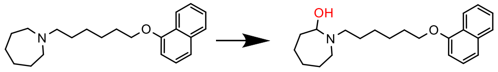
**23**	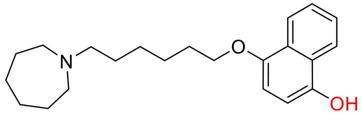	OC1=CC=C(C2=CC=CC=C12) OCCCCCCN3CCCCCC3	C22H31NO2	341,23548	Aromatic Hydroxylation of fused benzene ring	Enzyme: cytochrome P450 2D6 Biosystem: HUMAN	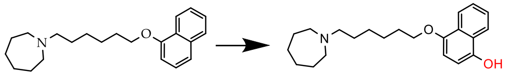
